# Ovarian Carcinosarcoma and Its Association with Mature Cystic Teratoma and Primary Tubal Carcinoma

**DOI:** 10.1155/2016/2605045

**Published:** 2016-10-11

**Authors:** Sunida Rewsuwan, Nopporn Satabongkoch, Prapaporn Suprasert, Surapan Khunamornpong

**Affiliations:** ^1^Department of Pathology, Faculty of Medicine, Chiang Mai University, Chiang Mai 50200, Thailand; ^2^Department of Obstetrics and Gynecology, Faculty of Medicine, Chiang Mai University, Chiang Mai 50200, Thailand

## Abstract

*Introduction*. Carcinosarcoma is an uncommon form of ovarian cancers, classified as being part of the group of mixed epithelial and mesenchymal tumors. The occurrence of carcinosarcoma in association with a mature cystic teratoma and synchronous tubal carcinoma is very rare.* Case Report*. A 69-year-old woman presented with a pelvic mass. An abdominal computerized tomographic scan detected a 15 cm right pelvic mass which was suggestive of malignant transformation of a dermoid cyst. Intraoperative, bilateral ovarian masses (left 10 cm and right 12 cm) with diffuse peritoneal metastatic nodules were identified. Histologically, the left ovarian mass was composed of 2 components including carcinosarcoma and mature cystic teratoma, whereas the right ovarian mass represented a mature cystic teratoma with serosal surface involvement of high-grade serous adenocarcinoma. The left fallopian tube was macroscopically unremarkable but contained a 5.0 mm focus of high-grade serous adenocarcinoma in the distal part, with adjacent serous tubal intraepithelial carcinoma.* Conclusion*. As the fallopian tube has recently been proposed to be an origin for a majority of pelvic or ovarian high-grade serous adenocarcinomas, tubal carcinoma may be the origin for ovarian carcinosarcomas through an epithelial-mesenchymal transition. The coexistence of ovarian carcinosarcoma and teratoma in the present case should represent a collision tumor.

## 1. Introduction

Carcinosarcoma is an uncommon type of ovarian cancers, accounting for 1–4% of patients [1–3]. Carcinosarcoma, also known as malignant mixed mullerian tumor, is a malignant neoplasm composed of epithelial carcinoma and malignant mesenchymal components and is classified as belonging to the group of ovarian mixed epithelial and mesenchymal tumors [4]. The current concept of the histogenesis of carcinosarcoma is that the sarcomatous component derives from the carcinomatous component through an epithelial-mesenchymal transition and metaplastic change of carcinoma cells [5].

Primary epithelial carcinoma of the tube accounts for only 0.1–1.8% of all gynecologic cancer cases [3, 6]. The incidence of tubal cancer was previously considered to be much less common than that of ovarian cancer. Recently, it has been proposed that the precursor lesion of tubal cancer, known as serous tubal intraepithelial carcinoma (STIC), is a possible origin for ovarian serous adenocarcinoma which is the most common histologic type of ovarian cancer [7].

The occurrence of ovarian carcinosarcoma in association with tubal carcinoma has rarely been described [1, 8]. To our knowledge, the combination of such coexistence and mature cystic teratoma (dermoid cyst) of the ovary has not been reported. We herein present a case in which ovarian carcinosarcoma coexisted with dermoid cyst and tubal carcinoma, the presentation of which mimicked advanced stage malignant transformation of an ovarian teratoma.

## 2. Case Report 

### 2.1. Clinical History

A 69-year-old postmenopausal woman (gravida 5, para 5) presented with a pelvic mass with weight loss for 1 month. She had a history of well-controlled hypertension for 6 years. Her body weight was 45.7 kg (BMI = 17.85 kg/m^2^). The physical examination revealed a suprapubic firm and movable mass involving the uterus and right adnexa. Serologic tumor markers were remarkable for the elevation of CA-125 (87.9 U/mL, normal 0–35) and CEA (14.6 ng/mL, normal 0–2.5), whereas the CA19-9 level was normal (20.38 U/mL, normal 0–37). An abdominal computerized tomography (CT) scan revealed a 15 × 9 cm right pelvic mass composed of enhanced solid tissue with a cystic component containing fat density and calcification, suggestive of malignant transformation of a dermoid cyst. Mild ascites was present. The other intra-abdominal organs were unremarkable.

An exploratory laparotomy was performed. Intraoperatively, bilateral ovarian masses were identified, left 10 cm and right 12 cm. There were generalized nodules in the peritoneal surface, including the uterus, cul-de-sac, urinary bladder, small and large intestine, omentum and mesentery, and the surface of the liver, spleen, and diaphragm. The patient underwent total abdominal hysterectomy, bilateral salpingooophorectomy, and partial omentectomy. There was a 5 cm residual tumor plaque in the cul-de-sac. The clinical diagnosis was FIGO stage IIIC ovarian cancer.

Postoperatively, the patient refused further treatment. Two months later, a large pelvic mass was detected with an elevation of serum CA-125 (277.1 U/mL). The patient accepted chemotherapy using paclitaxel and carboplatin. After completion of 6 cycles of chemotherapy, she developed bilateral cervical lymph node metastasis and ascites. The abdominal CT scan showed progression of intra-abdominal disease with a 4.4 cm metastatic lesion in the liver. She died of progressive disease 12 months postoperatively.

### 2.2. Pathological Findings

Macroscopically, the left ovarian mass was solid-cystic and composed of 4.5 cm cyst with a sebaceous content admixed with hairs connecting to a 5.5 cm multinodular solid mass. The right ovarian mass was solid-cystic composed of a unilocular cyst containing sebaceous material admixed with hairs and crescentic mural solid thickenings up to 5.5 cm in diameter. No macroscopic abnormality of either fallopian tube was observed. The uterus showed multiple serosal nodules without cervical or endometrial lesions. Multiple infiltrative nodules, up to 1.5 cm, were identified in the omentum.

**Figure 1:**
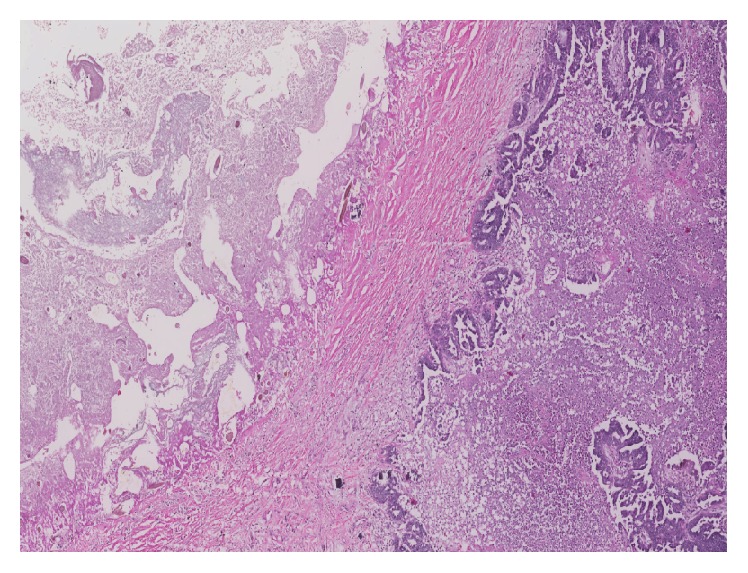
Left ovary: carcinosarcoma associated with the wall of a dermoid cyst with no demonstrable connection with internal cyst lining (hematoxylin and eosin, 40x).

**Figure 2:**
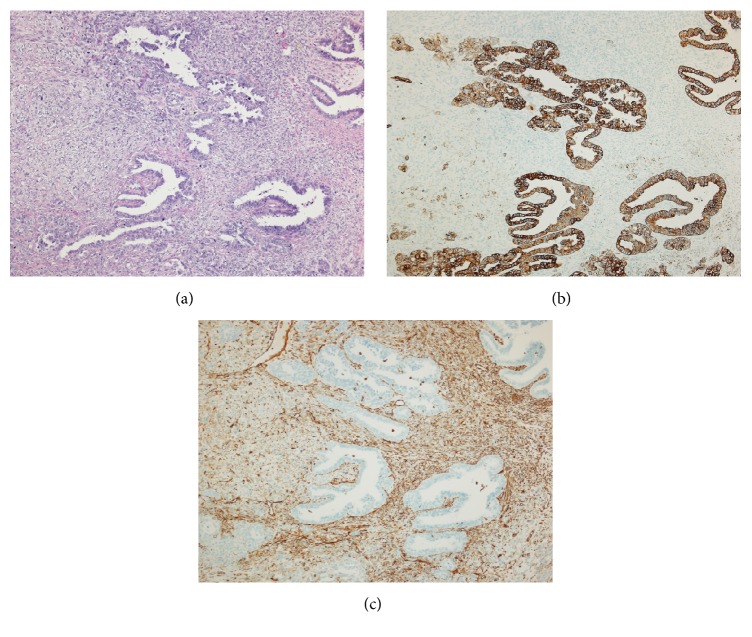
(a) Left ovary: carcinosarcoma consists of glands with complex papillary embedded in a solid sarcomatoid proliferation (hematoxylin and eosin, 100x). (b) Left ovary: the epithelial component displays strong reactivity indicating cytokeratin (AE1/AE3) (100x). (c) Left ovary: the sarcomatoid cell population illustrates focal staining for vimentin, while most of the epithelial component showed negative results (100x).

Histologically, the solid portion of the left ovarian mass was composed of an admixture of biphasic malignant components. The epithelial component was high-grade serous adenocarcinoma characterized by solid sheets and complex papillary architecture with slit-like and glandular arrangements. The neoplastic cells showed large round vesicular nuclei with prominent nucleoli and a high mitotic rate. The mesenchymal component was high-grade pleomorphic and spindle cell sarcoma of a nonspecified type (Figure 2(a)). The cystic component was a dermoid cyst composed of benign squamous epithelial lining with foci of cartilage, without connection between the cyst lining and malignant components (Figure 1). The right ovarian mass represented a dermoid cyst with serous adenocarcinoma involving the serosal surface. The peritoneal lesions were composed of serous adenocarcinoma only.

The left fallopian tube showed a 5.0 mm focus of high-grade serous adenocarcinoma up to 1.4 cm from the fimbriated end of the tube with invasion into tubal muscular wall. An adjacent focus of STIC was identified. There were no signs of any epithelial lesions in the right fallopian tube.

**Figure 3:**
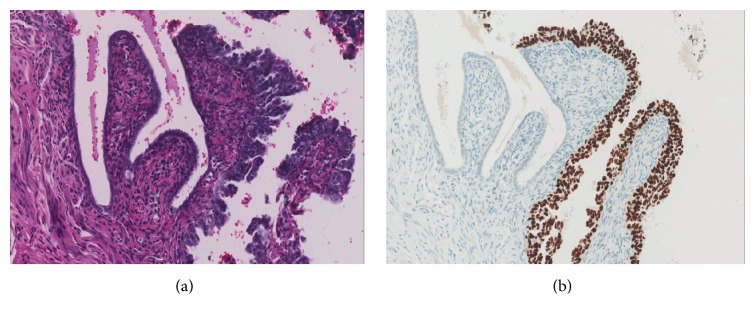
(a) Left fallopian tube: the serous tubal intraepithelial carcinoma (STIC) is noted in the tubal mucosa of the left fallopian tube (hematoxylin and eosin, 200x). (b) Left fallopian tube: the serous tubal intraepithelial carcinoma (STIC) displays strong nuclear p53 expression, in contrast to the adjacent tubal epithelia (200x).

The immunohistochemical studies gave positive reactions for cytokeratin (AE1/AE3) and cytokeratin 7 in the carcinomatous component (Figure 2(b)), whereas only a rare focal reaction was observed in the sarcomatous part. The sarcomatous component showed focal immunoreactions for vimentin, desmin, and actin, while the epithelial component was negative with these stains (Figure 2(c)). Both epithelial and mesenchymal components in the left ovarian tumor showed abnormal p53 expression (diffuse staining in over 90% of cells). The left tubal serous adenocarcinoma and adjacent STIC also showed a similar abnormal p53 expression (Figure 3).

## 3. Discussion

Synchronous malignant neoplasms in the female genital tract are rather uncommon but well recognized, accounting for 0.7–1.8% of patients with gynecological malignancies [9, 10]. Most of these represent synchronous endometrial and ovarian endometrioid adenocarcinoma of independent origin [9, 11]. Synchronous tubal carcinoma and endometrial or ovarian carcinoma is much less common [12].

It has recently been proposed that STIC may be an origin of high-grade serous adenocarcinoma of the tube, ovary, and peritoneum [8, 13]. The increasing recognition of STIC and the current protocol for extensive sectioning of fallopian tubes has led to the understanding that a fallopian tube may be the origin for ovarian carcinoma in a substantial number of cases. STIC was detected in association with ovarian serous carcinoma in 47% of cases of ovarian cancer [14]. The presence of STIC at the transition between benign tubal mucosa and invasive tubal serous adenocarcinoma and the location of carcinoma in the distal portion of tube, as observed in the present case, provides support for a primary tubal lesion. Carcinoma cells from the tube may implant in the ovary and further acquire sarcomatous phenotypes through the epithelial-to-mesenchymal transition and then transform into carcinosarcoma. An alternative explanation for ovarian carcinosarcoma in the present case may be a synchronous independent lesion by “field effect” carcinogenesis. Fortunately, the dilemma between the original tumor sites in this case was only of academic interest and did not significantly affect the management or treatment decisions.

Association of ovarian carcinosarcoma with contralateral serous adenocarcinoma of the ovary has been described in a 62-year-old woman by Vermes et al. [15]. The right ovarian serous cystadenocarcinoma was thought to originate from the superficial germinal epithelium, in contrast to the left ovarian carcinosarcoma which was considered to originate from the rudiments of the mullerian duct. The pathologic findings of both fallopian tubes were not mentioned. These neoplasms represented the presence of two distinct types of malignancy in the ovaries of the same patient. In another report by Arora et al., carcinosarcoma in the left broad ligament was associated with ipsilateral ovarian serous adenocarcinoma and endometrial endometrioid adenocarcinoma [16]. In that report, carcinosarcoma in the broad ligament was considered to represent the transformation of a metastatic lesion from the ovarian tumor. Brustman reported an association of ovarian carcinosarcoma with bilateral STIC in a 64-year-old woman. The patient had a 26 cm right ovarian carcinosarcoma which was composed predominantly of rhabdomyosarcoma with a minor component of mixed endometrioid and serous adenocarcinoma [1]. The relationship between STIC and ovarian carcinosarcoma has not been clarified.

The association of ovarian carcinosarcoma with a mature cystic teratoma has been reported in four previous cases [17–20], three of which had squamous cell carcinoma as the epithelial component, whereas one case had adenocarcinoma with signet ring differentiation. These cases were considered to be representing malignant transformation in dermoid cysts and transitional areas from benign epithelium to dysplastic epithelium. Invasive carcinoma was demonstrated in some cases. In the case described in this study, the carcinosarcomatous component did not show a demonstrable connection with the dermoid cyst lining, suggesting that they were coexisting or collision neoplastic lesions. In this patient, the radiologic findings had identified the presence of the teratomatous component which was suggestive of the possibility of a malignant transformation of a dermoid cyst. The serologic tumor markers also showed elevation of serum CEA with a relatively low rising of CA-125, which may be observed in the cases with such malignant transformation [21] but was rather uncommon in the patients with advanced stage ovarian epithelial carcinoma.
